# *Dead-End* (*dnd*) Gene Cloning and Gonad-Specific Expression Pattern in Starry Flounder (*Platichthys stellatus*)

**DOI:** 10.3390/ani11082256

**Published:** 2021-07-30

**Authors:** Ji-Hye Yoon, Youn-Su Cho, Hyo-Bin Lee, Jung-Yeol Park, Han-Kyu Lim

**Affiliations:** 1 Department of Interdisciplinary Program of Biomedicine, Health & Life Convergence Science, Mokpo National University (MNU), Muan 58554, Korea; bdh00258@naver.com (J.-H.Y.); leeehyobin@gmail.com (H.-B.L.); 2 Department of Fishery Biology, Pukyong National University (PKNU), Busan 48512, Korea; whdbstn12@gmail.com; 3 Department of Marine and Fisheries Resources, Mokpo National University (MNU), Muan 58554, Korea; jungyeol89@naver.com

**Keywords:** *dead end*, PGC, germ cell, maternal gene, *Platichthys stellatus*

## Abstract

**Simple Summary:**

The *dead end* (*dnd*) gene encodes an RNA-binding protein that plays a role for migration of primordial germ cells (PGCs) to the gonadal region during embryogenesis in vertebrates. Here, the starry flounder (*Platichthys stellatus*) *dead end* (*psdnd*) gene was characterized and its expression patterns were analyzed. Full-length *psdnd* mRNA was 1495 bp long, encoding 395 amino acids. *psdnd* was only expressed in gonadal tissues, we detected no *psdnd* expression in somatic cells. Furthermore, *psdnd* was strongly expressed during early embryogenesis. Our findings suggest that *psdnd* expression is gonad-specific and could therefore be used as a germ cell marker in starry flounder.

**Abstract:**

*dnd* is a germline-specific maternal RNA expressed in various vertebrate classes, which encodes an RNA-binding protein that is essential for PGC migration. The purpose of this study is fundamental research about starry flounder *dnd* gene for germ cell marker development. In this study, we cloned and analyzed the expression levels of *Platichthys stellatus dead end* (*psdnd*) in various tissues and embryonic stages. The *psdnd* gene was isolated from starry flounder ovaries, cloned into a pGEM-t vector, and sequenced. Full-length of *psdnd* cDNA was 1495 bp long, encoding 395 amino acids. *psdnd* expression levels were investigated by real-time polymerase chain reaction (qRT-PCR) in various tissues and embryo developmental stages. *psdnd* transcripts were detected in the testes and ovaries, but not in somatic tissues. Embryonic *psdnd* expression levels were higher during early embryo development stages than during late embryogenesis; *psdnd* expression was highest at the 1 cell stage, then gradually decreased throughout the subsequent developmental stages. The spatial expression pattern was analyzed by whole-mount in situ hybridization (WISH). The *psdnd* transcripts migration pattern was similar with zebrafish (*Danio rerio*). Our results suggest that *psdnd* may function as a germ cell-specific marker.

## 1. Introduction

PGCs are the only cells that can transmit genetic material to the next generation. PGCs move to the gonadal location during early embryogenesis where they develop into gonads [1]. The proliferation of PGCs become the germline stem cells which can develop to mature gonads. Furthermore, migration of PGCs impacts on the fertility [2]. The cell fate and development of PGCs are determined by germ plasm mRNA derived from the maternal line [2]. Maternal germplasm mRNAs encode evolutionarily conserved proteins, including *vasa*, *nanos C2HC-type zinc finger 3* (*nanos3*), *dead-end* (*dnd*), *tudor-domain-containing protein* 7 (*tdrd7*), and *deleted in azzospermia-like* (*dazl*) [2,3,4,5,6].

*dnd* is a germ plasm-specific marker in several vertebrate species that encodes an RNA-binding protein essential for PGC migration [7]. Uracil-rich sequences in the microRNA 430 bind to DND in zebrafish (*Danio rerio*). *dnd* prevents the degradation of germ plasm mRNAs targeted by mi430, thus maintaining germ cell development [8].

Germ cell-specific expression of *dnd* was first discovered in zebrafish after which similar expression patterns were found in various vertebrate species including mice (*Mus musculus*), frogs (*Xenopus tropicalis*), chickens (*Gallus gallus*), and humans (*Homo sapiens*) [8,9,10,11]. Thus, *dnd* is an evolutionarily conserved gene. In zebrafish and medaka (*Oryzias latipes*), germ cells are essential for female gonadal differentiation and the absence of PGCs induces sterility in males [12,13]. In addition, the removal of PGCs from pond loach (*Misgurnus anguillicaudatus*) and goldfish (*Carassius auratus*) induces sterility both males and females [14,15]. Abnormal PGC localization was induced in zebrafish by knocking down *dnd* expression [12]. In frogs, PGCs were lost when *dnd* was knocked down using morpholino oligonucleotide [16]. These studies indicate that *dnd* is essential for germ cell development during embryogenesis.

Most farmed fish reach gonadal maturation before they are released to the market, and gonadal maturation is an important factor that determines the marketability of fish [17]. However, infertile fish exhibit improved growth rates, meat quality, and disease resistance; these effects are achieved by minimizing the energy required for gonadal development [18,19,20,21,22]. In addition, sterilization is an effective method to reduce ecological genetic contamination by protecting natural species from escaped farmed fish, thus maintaining genetic diversity [17].

The development of sterilization strategies is an important goal for researchers. To achieve this, genetic modification techniques such as chromosome manipulation, transgene expression, morpholino oligonucleotide treatment, and CRISPR/Cas9 are increasingly undergoing development and application in the aquaculture industry. *dnd* knockdowns can induce sterility in organisms by blocking PGC migration to the gonad developmental region. Genetic engineering techniques have been applied to knock-down *dnd* in channel catfish (*Ictalurus punctatus*) [23]. Furthermore, salmon were successfully sterilized by targeting *dnd* with CRISPR/Cas9 [24]. Infertility was induced in sterlet (*Acipenser ruthenus*) by knocking down *dnd* with an antisense morpholino oligonucleotide [25].

To support novel technologies for producing infertile eggs in starry flounder, we conducted fundamental research including identification of *psdnd* and expression pattern during embryogenesis. Improving our understanding of the mechanisms driving gonad development, such as *dnd* expression, would enable the development of new sex maturation control technologies.

## 2. Materials and Methods

### 2.1. Animals and Sampling

3 females and 3 males which fully matured starry flounders were used in this experiment from Marine seed fish farm (Yeosu, Korea). These fishes were raised in a 15 ton tank controlling the water temperature. The mean total length and weight of females was 33.8 ± 2.8 cm and 825.7 ± 186.5 g. The mean total length and weight of males was were 32.6 ± 0.9 cm and 504.6 ± 87.4 g. Starry flounders were kept on 16:8 light:dark cycle. Exogenous salmon gonadotropin-releasing hormone analog (sGnRHa) (Ovaplant, Syndel, Ferndale, WA, USA) pellets were inserted into the dorsal muscles at a concentration of 50 µg/kg. Various tissues including brain, gill, heart, kidney, eye, stomach, gut, spleen, liver, muscle, testis, and ovary were collected from fully matured female and male starry flounders. Fertilized eggs were cultured at 10 ± 1 °C after artificial insemination. Embryo samples including unfertilized egg, 1 cell, 2 cell, 4 cell, 8 cell, 16 cell, morula, blastula, early gastrula, late gastrula, somite, and hatching larva were collected for analysis. Tissues and embryos were stored in liquid nitrogen prior to RNA extraction.

### 2.2. Total RNA Extraction and cDNA Synthesis

Total RNA was extracted followed by the manufacturer’s instructions. Total RNA was extracted from 100 mg of each tissue (brain, gill, heart, kidney, eye, stomach, gut, spleen, liver, muscle, testis, and ovary) using TRIzol® (Invitrogen, Waltham, MA, USA). In addition, total RNA was extracted from 100 mg of embryo tissue at 12 distinct developmental stages using TRIzol® reagent. 200 μL of chloroform were added and reacted at −20 °C for 5 min. Centrifugation at 12,000 rpm, 4 °C for 15 min. The supernatant was mixed with the same amount of isopropanol and reacted for 10 min. Centrifuge at 12,000 rpm, 4 °C for 10 min. The supernatant was removed and 1 mL of 70% ethanol was added for washing the pellet. The ethanol was removed completely by centrifuging at 12,000 rpm, 4 °C for 5 min. 100 μL of diethylpyrocarbonate (DEPC) water was added to the pellet and resuspended. DNase I (Qiagen, Hilden, Germany) was treated to prevent DNA contamination. cDNA was synthesized from purified total RNA using a Maxima First Strand cDNA Synthesis Kit (Thermo, Waltham, MA, USA). Two microliters of Maxima Enzyme Mix, 4 μL of 5 × reaction mix, and 14 μL of RNA were mixed for real-time PCR. The cDNA synthesis conditions were 25 °C for 10 min, 65 °C for 30 min, and 85 °C for 5 min. The synthesized cDNA was quantified using a NANODROP ONEC (Thermo, Waltham, MA, USA) and stored in a deep freezer (−80 °C).

### 2.3. PCR Amplification of cDNA Fragment

Primers were designed based on the *dnd* mRNA sequence of olive flounder to detect and amplify *psdnd*. The olive flounder *dnd* sequence (Accession No. KP224455.1) was obtained from the National Center for Biotechnology database (Table 1).

The PCR cycling conditions were as follows: pre-denaturation at 94 °C for 5 min; 30 cycles of 94 °C for 30 s, annealing at 55 °C for 30 s, and extension at 72 °C for 2 min; then a final extension at 72 °C for 10 min. The PCR products were analyzed by electrophoresis with a 1% agarose gel. The DNA was gel extracted and sequenced by Macrogen Inc. (Seoul, Korea).

### 2.4. 5′ and 3′ Rapid Amplification of cDNA Ends (RACE) PCR

5′ and 3′ gene-specific primers (GSPs) were designed based on the partial fragment of starry flounder *dnd* to obtain the full transcript (Table 1). 5′ RACE PCR was conducted using a 5′ RACE system for Rapid Amplification of cDNA Ends kit version 2.0 (Invitrogen, Waltham, MA, USA), in accordance with the manufacturer’s instructions.

3’ cDNA was synthesized using a SMART cDNA synthesis kit (Clontech, MountainView, CA, USA). Total RNA was isolated from starry flounder ovarian tissues using TRIzol® reagent. Single-step PCR and semi-nested PCR were performed using the synthesized 3’ cDNA. One microliter of 3’ GSP1 primer, 1 µL of universal primer mix, and 2 μL of 3’ cDNA were added to the reaction mixture containing 0.25 μL of Advantage Taq polymerase (Takara, Nojihigashi, Japan), 1.5 μL of 10 × buffer, 1 μL of dNTPs, and sterile water to a total volume of 10 μL. The single-step PCR cycling conditions were as follows: pre-denaturation at 94 °C for 2 min 30 s; 35 cycles of 94 °C for 30 s, annealing at 58 °C for 30 s, and amplification at 72 °C for 2 min; then a final extension at 72 °C for 5 min. The semi-nested PCR was conducted with 1 μL of nested universal primer, 1 μL of 3’ GSP2, 1 μL of the single-step PCR product, 17 μL of 1.1 × master mix, and sterile water to a total volume of 20 μL. The semi-nested PCR was performed using thermocycling conditions identical to the conditions used for single-step PCR. PCR products were an-alyzed by means of electrophoresis and sequencing, performed by Macrogen Inc. (Seoul, Korea).

### 2.5. Cloning

Primers were designed based on the 1525 bp *psdnd* cDNA sequence obtained via 5’ and 3’ RACE PCR. One microliter of ovary cDNA was amplified with AccuPower^®^ PCR premix (Bioneer, Deajeon, Korea) and 10 pM of each primer (sense and antisense). The PCR cycling parameters were as follows: initial denaturing at 94 °C for 5 min; 35 cycles of denaturing at 94 °C for 30 s, annealing at 53 °C for 30 s, and elongation at 72 °C for 2 min; then a final elongation at 72 °C for 10 min. PCR products were analyzed on a 1% agarose gel and DNA within a size range of 1400–1600 bp was extracted using a Gel extraction kit (Bioneer, Korea). cDNA (30 ng/μL) was ligated into the pGEM-T vector system I (Promega, Madison, WI, USA), in accordance with the manufacturer’s instructions. Ten microliters of ligation mixture were used to transform 50 μL of *Escherichia coli* DH5α Competent Cells (Takara, Nojihigashi, Japan), and the cells were incubated at 37 °C for recovery. The transformation culture was then incubated for 16 h at 37 °C on MacConkey agar supplemented with ampicillin for colony selection. Plasmid DNA was extracted using an Accuprep® plasmid Mini extraction kit (Bioneer, Korea). The extracted plasmid DNA was sequenced by Macrogen Inc. (Seoul, Korea).

### 2.6. Phylogenetic Analysis of psdnd

Molecular phylogenetic analysis was performed to compare the *dnd* sequences of different vertebrate species. The full *dnd* gene sequences of various species were retrieved from the NCBI database. The GenBank accession numbers of the sequences examined were as follows: atlantic salmon (*Salmo salar*), JN712911.1; channel catfish (*Ictalurus punctatus*), XM_017484732.1; chicken (*Gallus gallus*), XM_015293536.2; common carp (*Cyprinus carpio*), XM_019103334.1; atlantic halibut (*Hippoglossus hippoglossus*), XM_034598806.1; human (*Homo sapiens*), NM_194249.3; pond loach (*Misgurnus anguillicaudatus*), MH283870.1; mouse (*Mus musculus*), NM_173383.2; olive flounder (*Paralichthys olivaceus*), KP224455.1; pacific bluefin tuna (*Thunnus orientalis*), KF128758.1; rainbow trout (*Oncorhynchus mykiss*), MK887177.1; turbot (*Scophthalmus maximus*), KC460339.1; western clawed frog (*Xenopus tropicalis*), NM_001044434.1 and zebrafish (*Danio rerio*), AY225448.1.

A phylogenetic tree was constructed with the Mega X version 10.1 software using the maximum-likelihood method with a bootstrap analysis of 1000 replicates. The percentage identities between the starry flounder *dnd* sequence and the homologues genes from other species were analyzed with CLUSTALW.

### 2.7. qRT-PCR

qRT-PCR was performed using a Pikoreal 96 RealTime PCR System (Thermo, Waltham, MA, USA) to examine the *psdnd* expression profiles in various tissues and stages of embryo development. The concentrations of cDNA from eight distinct tissues and 12 developmental stages were adjusted to 50 ng/μL and 500 ng/μL respectively. The primers used for qRT-PCR are listed in Table 1. *dnd* transcripts were detected using SYBR Green PCR Mastermix (Thermo, Waltham, MA, USA), mixed with 1 μL of each primer (10 pM), 1 μL of cDNA, and 7 μL of sterile water. The qRT-PCR cycling conditions were as follows: pre-incubation at 50 °C for 2 min; pre-denaturation at 95 °C for 7 min; and then 40 cycles of denaturation at 95 °C for 15 s, annealing at 55 °C for 30 s, and extension at 72 °C for 20 s. Expression levels of the housekeeping gene glyceraldehyde3-phosphate dehydrogenase were used to calculate the relative expression levels of *psdnd*. *psdnd* expression levels in the spleen and fertilized eggs were used to calculate Ct values. Relative expression levels were determined by calculating 2^−ΔΔCt^ using mean Ct values. The qRT-PCR primer sequences are shown in Table 1.

### 2.8. Whole Mount In Situ Hybridization (WISH)

Digoxigenin-labeled antisense probes were synthesized from linearized plasmids containing the *dnd* sequence using a DIG RNA Labeling kit (Roche, Basel, Switzerland). The primers used for WISH are shown in Table 1. Embryo samples were fixed in 4% paraformaldehyde with phosphate-buffered saline. The embryo samples were dehydrated using 50% and 30% methanol, and the pigments were removed from the embryo samples using 6% hydrogen peroxide and 0.5% potassium hydroxide. Then, proteinase K solution (10 μg/mL) was applied to permeabilize embryo membranes, and the embryo samples were fixed again in 4% paraformaldehyde. Samples were incubated in hybridization solution at 65 °C for 3 h; 1 μg/mL antisense RNA probe was then added to the hybridization solution and incubated at 85 °C for 3 minutes. A longer hybridization reaction was also conducted at 65 °C for 12 h. The *dnd* transcripts were visualized using purple alkaline phosphatase substrate.

### 2.9. Statistical Analysis

The qRT-PCR results were expressed as mean ± SD. Statistical analyses were performed using SPSS Statistic version 26.0 (SPSS Inc., Chicago, IL, USA). One-way analysis of variance and Tukey’s test were used to determine significance (*p* < 0.05).

## 3. Results

### 3.1. Identification of P. stellatus cDNA Sequence

**Figure 1:**
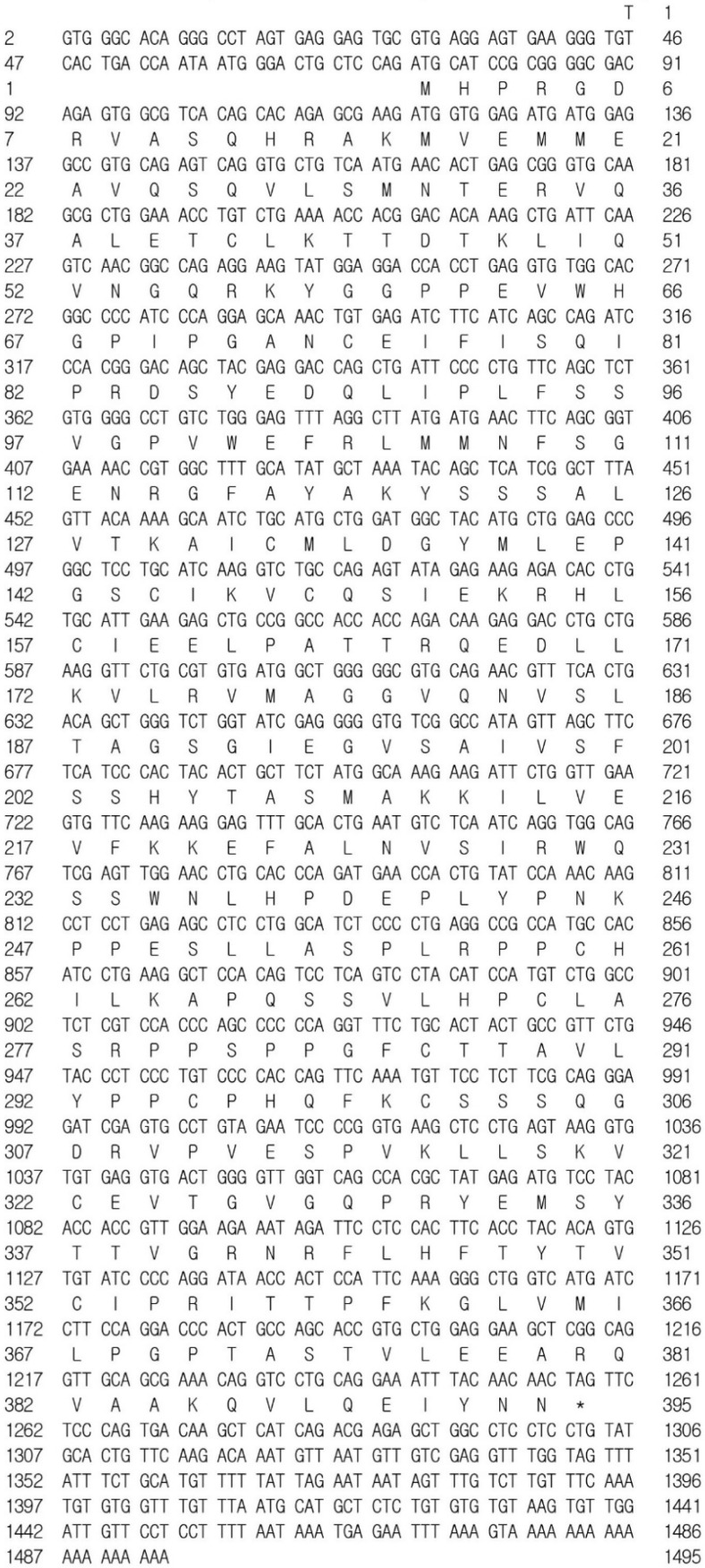
Nucleotide and amino acid sequence of *psdnd.* The first methionine amino acid indicates the start codon. Strock indicates the stop codon. Amino acid sequence was analyzed by clustal omega website available at https://www.ebi.ac.uk/Tools/msa/clustalo/ (accessed on 15 March 2020).

**Figure 2:**
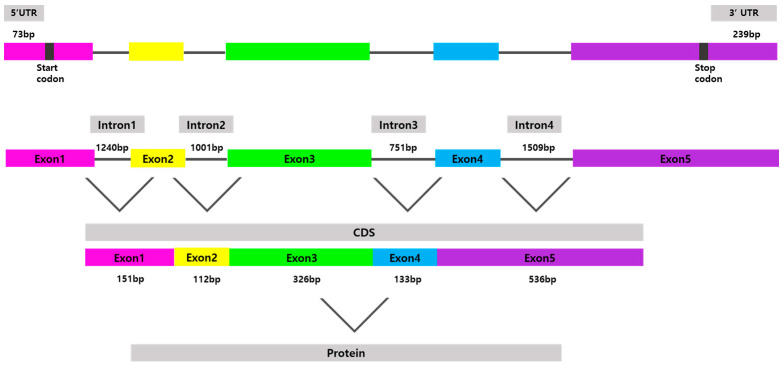
Physical map of cloned *psdnd.* The *psdnd* open reading frame (ORF) contains 5 exons and 4 introns. Revealed 73 bp of 5′UTR and 239 bp of 3′UTR. Each exons are shown in different color boxes. Introns expressed thin grey lines.

The full-length *psdnd* mRNA was 1495 bp long, containing an 1185 bp coding sequence encoding 395 amino acids (Figure 1). *psdnd* cDNA had five exon regions; the length of exons 1 to 5 were 151 bp, 111 bp, 327 bp, 133 bp, and 536 bp respectively. The length of the 5′-untranslated region was 73 bp. The 3′-untranslated region was 239 bp long, including the complete 3′ end with a poly-(A)-tail. *psdnd* genomic DNA had 4 introns with lengths of 1240 bp, 1001 bp, 751 bp, and 1509 bp (Figure 2). 

### 3.2. Multiple Sequence Alignments

**Figure 3:**
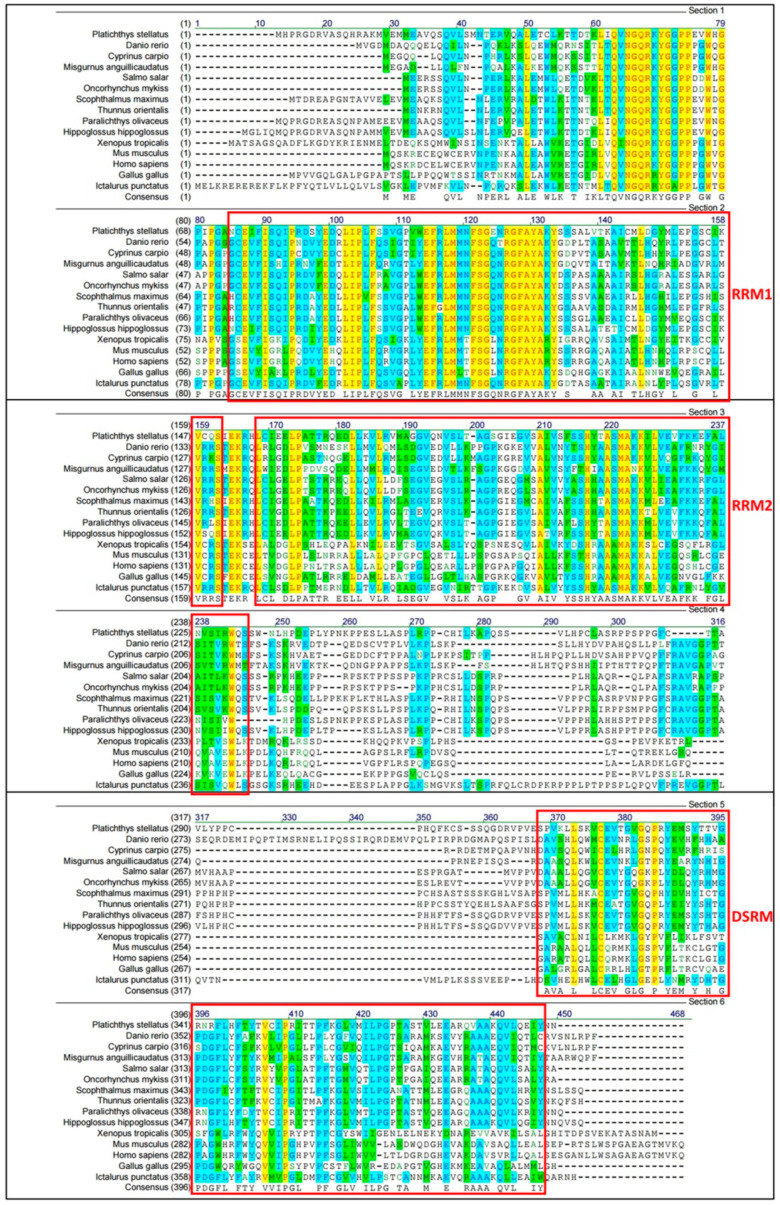
Multiple alignments of *psdnd* with *dnd* homologues from various vertebrate species. Black text on white: non-similar residues. Blue text on cyan: consensus residue, derived from a block of similar residues at a specific position. Black text on green: consensus residue, derived from an occurrence of ≥50% of a single residue at a specific position. Red on yellow: consensus residue, derived from a completely conserved residue at a specific position. Green text on white: residue similar to consensus residue at a specific position. Red boxes indicate conserved motifs including RRM1, RRM2 and, DSRM. Multiple alignment analyzed by align X program. Conserved domain was analyzed at NCBI conserved domain site.

A multiple alignments comparing the amino acid sequence deduced from *psdnd* with the *dnd* sequences of other species is shown in Figure 3. The range of sequence identities of the *dnd* amino acid sequences was 31–85% (Table 2). Of the alignment, data suggested that several regions of the *dnd* protein were conserved between species. The amino acid sequence determined using the *psdnd* cDNA sequence possessed three conserved motifs including two RNA-recognition motifs (RRMs) and one double-stranded RNA-binding motif (DSRM). The RRMs and DSRM were shared among *dnd* homologues.

### 3.3. Phylogenetic Tree and Identity

**Figure 4:**
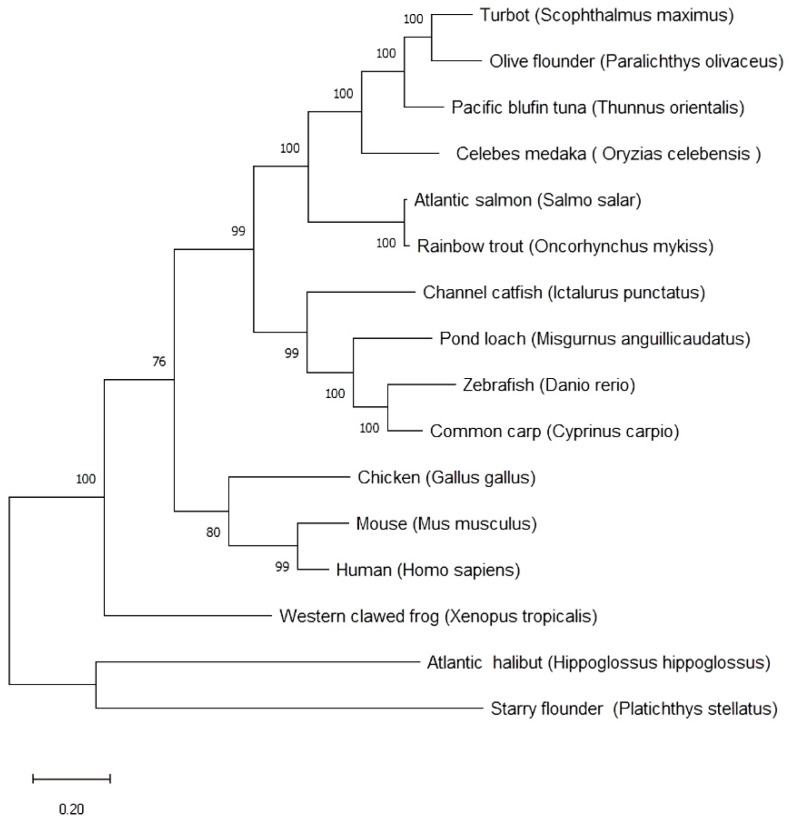
Phylogenetic tree and sequences identities comparing *psdnd* with *dnd* from various vertebrate species. Phylogenetic tree of *psdnd* established by MEGAX program with maximum likelihood method. The numbers under the nodes indicate bootstrap percentage values for 1000 replicates. Scale bar indicates number of substitution changes per site. Bootstrap values indicate branch.

The deduced *psdnd* amino acid sequence was aligned with related protein sequences from various vertebrate species (Table 2). According to the neighbor-joining tree shown in Figure 4, *psdnd* and atlantic halibut *dnd* formed a clade supported by a bootstrap value of 100%. *psdnd* exhibited identities of 85%, 77%, 62%, 61%, 50%, 49%, 39%, 39%, 37%, 35%, 33%, 32%, 32%, and 31% with *dnd* homologues in atlantic halibut, olive flounder, turbot, pacific bluefin tuna, rainbow trout, Atlantic salmon, pond loach, channel catfish, common carp, zebrafish, human, mouse, chicken, and western clawed frog respectively (Figure 4).

The highest protein sequence identity is 85% of Atlantic halibut. As followed DND protein identity ranked high 77% of olive flounder and 62% of turbot. The lowest protein sequence identity is 31% of western clawed frog.

### 3.4. qRT-PCR

**Figure 5:**
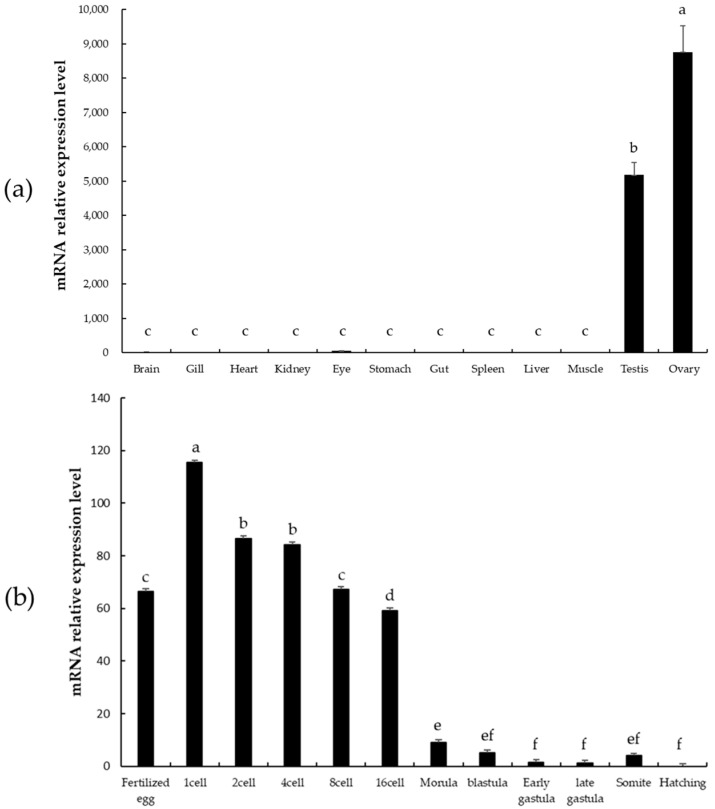
*psdnd* transcript expression levels in various tissues and embryo stages measured by qRT-PCR. (**a**) Tissue-specific expression of *psdnd* (*p* < 0.05). (**b**) Embryonic expression of *psdnd.* Different letters indicate statistically significant difference (*p* < 0.05).

*psdnd* transcripts were detected in both male and female gonads by means of qRT-PCR. However, *psdnd* expression was significantly greater in the ovaries than in the testes. No *psdnd* expression was detected in the other tissues examined (Figure 5a).

*psdnd* mRNA was expressed during all stages of embryonic development and expression was maintained until hatching (Figure 5b). Strong expression was detected from the fertilized egg stage until the 16 cell stage. After fertilization, *psdnd* transcript expression increased and peaked at the 1 cell stage. *psdnd* expression decreased slightly until 16 cell stage, then decreased dramatically from the 16 cell stage to the hatching stage.

### 3.5. Whole-Mount In Situ Hybridization

**Figure 6:**
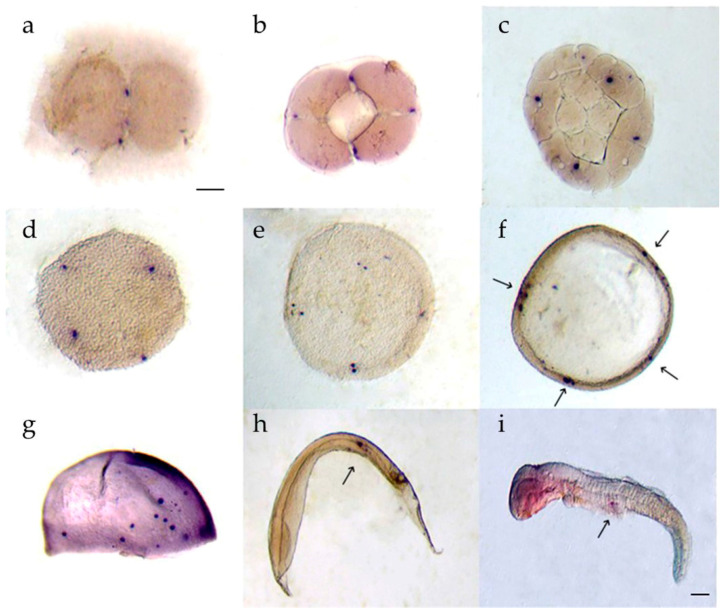
*psdnd* transcript localization during embryogenesis. Embryos were hybridized with antisense *psdnd*, stained by purple AP. (**a**) 2 cell stage: *psdnd* transcripts presented at first cleavage site; (**b**) 4 cell stage: *psdnd* positive signals revealed the spots where edges of second cleavage; (**c**) 32 cell stage; (**d**) morula stage: four *dnd* positive signals maintained until morula stage; (**e**) blastula stage: sister cells developed next to origin cells; (**f**) early gastrula stage; *dnd* transcripts increased and clustered at four spots of germ rings; (**g**) late gastrula stage; *psdnd* positive signals were move on to body axes; (**h**) early somite stage; (**i**) hatching larva: *psdnd* transcripts were aggregated and move on to future gonad location. Arrows indicate *psdnd* transcript expression. Scale bar: 100 μm.

Two spots of *dnd* positive cells were detected at the edges of the first furrows during the 2 cell stage (Figure 6a). *psdnd* was expressed in four spots at the ends of the second furrows from the 4 cell to the morula stage (Figure 6b–d). At the blastula stage, multiple sister cells were generated close to the *psdnd* positive-cells (Figure 6e). During the early gastrula stage, *psdnd* expression was detected at the blastoderm margins (Figure 6f). At the late gastrula stage, few *psdnd* transcripts were dispersed however they were on move at the trunk mesoderm (Figure 6g). The *psdnd* positive signals then moved toward the dorsal mesentery, where the gonads would later develop; *psdnd* expression continued in this area until the hatching stage (Figure 6h,i).

## 4. Discussion

In this study, the *psdnd* protein sequence contains two RRM motif and one DSRM motif at its C-terminus, which are characteristic of DND protein [1]. The interspecific similarities in the DND amino acid sequences suggest that they have similar structures and functions, and that DND is well conserved. The first RRM motif, RRM1, is the main binding site for DND1 protein that directly binds to nanos1. The second RRM motif, RRM2, plays a role in promoting nanos1 translation and germline survival in xenopus [26]. DSRM is not essential for RNA binding, although it increases the target specificity of the RRM regions in DND1 in human [27]. Zebrafish *dnd* has a binding site to bind miR-430 for preventing degradation from miRNA [8]. However, the structure and function of which has not been evaluated in starry flounder. Further study is needed to understand the relationship between *psdnd* and miR-430 in starry flounder.

The *psdnd* protein sequence exhibited strong homologues with DND proteins of other species, especially other flatfish species such as Atlantic halibut (85%), olive flounder (77%), and turbot (62%). The phylogenetic tree and identity results indicated that *dnd* in starry flounder and Atlantic halibut have the highest evolutionary closeness in our researched target species.

The transcript expression of *psdnd* was analyzed in various tissues by means of qRT-PCR. *psdnd* expression was only detected in the ovaries and testes, suggesting that *psdnd* can be used as a germ cell marker in starry flounder. In some species, such as mouse and xenopus, *dnd* expression is sex-dependent [13,28]. However, in fish species, *dnd* is expressed in both sexes, as observed in this study [7,13,18,29,30]. The expression in both ovary and testis means *psdnd* has specific roles for oogenesis and spermatogenesis during sexual maturation in starry flounder.

The expression levels of *psdnd* at various embryo developmental stages were also investigated by qRT-PCR. We found that *psdnd* was highly expressed during the early developmental stages, from the 1 cell to the early gastrula stage. Higher expression patterns during early embryogenesis also revealed in olive flounder and turbot [7,28]. This suggests that embryonic *psdnd* expression is of maternal origin, similar with the germplasm marker, *vasa. psdnd* was also detected at the zygote stage. After the gastrula stage, *psdnd* transcripts began to decrease more dramatically. Maternal genes are often highly expressed until the zygotic genome is activated. Depending on the genes or species, continuously sex-pressed gene can interact with the zygotic genome [31]. The pattern of *psdnd* expression pattern may be related to the expression of the microRNA 430. MicroRNAs are small molecules that regulate the expression of target mRNAs by degradation. In zebrafish, miR-430 targets *nanos* and *tdrd7*, which are essential for PGC development. DND1 prevents miR-430 from binding to its target mRNAs. After zygotic genome activation at the gastrula stage, miR-430 and DND1 expression levels decrease [8,32,33]. In this study, we found that *psdnd* was strongly expressed before the blastula stage, suggesting that *psdnd* was of maternal origin.

The successful migration of PGCs to the gonads is essential for PGC development and survival. PGCs require maternally supplied germplasm and mRNAs for their specification and maintenance. Our WISH results revealed that the *psdnd* expression pattern was similar with the PGC localization pattern, suggesting that *psdnd* is a germplasm involved in PGC migration. Similar quantitative and spatial expression patterns occur for *dnd* in other vertebrate species, including olive flounder, turbot, and rare minnow [7,30,31].

This finding of this study suggests that *psdnd* could be used as a germ cell marker in starry flounder; moreover, *dnd* could be a potential target for starry flounder sterilization.

## 5. Conclusions

In this study, we identified and cloned full-length *psdnd*, which was 1495 bp long and encoded 395 amino acids. *psdnd* was specifically expressed in starry flounder ovaries and testes. Moreover, *psdnd* was highly expressed during the early embryonic developmental stages, from fertilized egg to the 16 cell stage, after which expression significantly decreased. Our results suggest that *psdnd* is maternally derived and specifically expressed in germ cells, similar with *dnd* homologs in organisms.

## Figures and Tables

**Table 1:** Primer and probe sequences for *psdnd* cloning and qRT-PCR.

Purpose	Primer Name	Primer Sequence (5′-3′)	Product Size_(bp)
Partial fragment	*dnd*-F	ATGAACACTGAGCGGGTGCAAGC	1098
	*dnd*-R	CTAGTTGTTGTAAATTTCCTGCAGG	
5′ RACE	5′ GSP1	AGATTGCTTTTGTAAC	≥238
	5′ GSP2	AGACAGGCCCCACAGAGCTGAAC	≥145
	5′ GSP3	AGCTGGTCCTCGTAGCTGTCCCGTG	≥113
3′ RACE	3′ GSP1	GCGTGCAGAACGTTTCACTGACAGCTGGG	≥647
	3′ GSP2	TGCCACATCCTGAAGGCTCCACAGTCCTC	≥408
Cloning	CL-F	GCGTGCAGAACGTTTCACTGACAGCTGGG	1493
	CL-R	TGCCACATCCTGAAGGCTCCACAGTCCTC	
qRT-PCR	*dnd*-qF	AGAACGTTTCACTGACAGC	100
	*dnd*-qR	AGAACGTTTCACTGACAGC	
qRT-PCR	GAPDH-qF	CCAGAACATCATCCCAGCTT	185
	GAPDH-qR	GGCCTTCACAACCTTCTTGA	
WISH	WISH-F	AGATCCCACGGGACAGCTAC	761
	WISH-R	GTTGGTCAGCCACGCTATGAG	

**Table 2:** Sequence identities comparing *psdnd* to homologues in other species.

Species	Identity (%)
Starry flounder (*Platichthys stellatus*)	100
Atlantic halibut (*Hippoglossus hippoglossus*)	85
Olive flounder (*Paralichthys olivaceus*)	77
Turbot (*Scophthalmus maximus*)	62
Pacific blufin tuna (*Thunnus orientalis*)	61
Rainbow trout (*Oncorhynchus mykiss*)	50
Atlantic salmon (*Salmo salar*)	49
Pond loach (*Misgurnus anguillicaudatus*)	39
Channel catfish (*Ictalurus punctatus*)	39
Common carp (*Cyprinus carpio*)	37
Zebrafish (*Danio rerio*)	35
Human (*Homo sapiens*)	33
Mouse (*Mus musculus*)	32
Chicken (*Gallus gallus*)	32
Western clawed frog (*Xenopus tropicalis*)	31

## Data Availability

All data are contained within the article.
